# Seroprevalence of Measles Antibodies and Predictors for Seropositivity among Chinese Children

**DOI:** 10.3390/ijerph14060605

**Published:** 2017-06-06

**Authors:** Xiaoqin Wang, Mei Ma, Zhaozhao Hui, Paul D. Terry, Yue Zhang, Rui Su, Mingxu Wang, Wei Gu, Ling Li

**Affiliations:** 1 Department of Public Health, Xi’an Jiaotong University Health Science Center, Xi’an 710061, China; wangxiaoqin@mail.xjtu.edu.cn (X.W.); wysun201314195@163.com (M.M.); huizhaozhao93@163.com (Z.H.); zymoon95@126.com (Y.Z.); 232guwei@xjtu.edu.cn (W.G.); liling_ch@163.com (L.L.); 2 Department of Medicine, University of Tennessee Medical Center, Knoxville, TN 37920, USA; pterry@utmck.edu; 3 Center for Disease Control and Prevention in Qian County, Xianyang 713300, China; rishi113@163.com

**Keywords:** seroprevalence, measles, vaccine dose, predictor

## Abstract

*Background*: Supplementary measles immunization has been implemented since 2010 throughout China, yet few studies have reported its effect in the northwest regions. *Methods:* A cross-sectional study was conducted among children aged 2 to 4 years old (*n* = 755) from February to September 2014 in 25 towns of Qian County, Shaanxi Province. Blood samples were analyzed for measles antibodies using enzyme-linked immunosorbent immunoglobulin G (IgG) assays. Socio-demographic factors were assessed by questionnaire. Data on vaccine dose were collected from town medical records. Univariate and logistic regression analyses were used to determine factors associated with measles antibody seropositivity. *Results*: Measles antibody seroprevalence was 91.13% (95% CI: 89.52–92.83) in our sample. Compared with children whose mother’s highest education was primary school, seroprevalence was higher in children whose maternal education was middle school (adjusted OR: 1.4, 95% CI: 0.7–2.8), high school (adjusted OR: 2.4, 95% CI: 1.3–7.7), and college/university (adjusted OR: 2.9, 95% CI: 1.2–9.3). Vaccine dose was positively associated with seropositivity. *Conclusions*: Measles seroprevalence is high in China and is associated with the mother’s education and vaccine dose.

## 1. Introduction

Measles is a highly contagious infectious airborne disease caused by morbillivirus [1] that usually occurs in children and non-immune people of any age. It affects about 20 million people per year [2], leading to 134,200 deaths globally in 2015, primarily in the developing areas of Africa and Asia [1]. As of 20 January 2017, China ranked fifth in the World Health Organization’s (WHO) western pacific region with an annualized measles incidence rate of 17.4 per million people [3].

From 1986 to 2005, Chinese health authorities mandated that children should be vaccinated with two doses of measles vaccine, given at 8 months and 7 years of age [4]. A WHO position paper suggests that the people are protected after 2 vaccine doses even when antibodies are below the protective level due to immune memory [5]. Even though the Chinese government has made great efforts to reduce morbidity and mortality from the disease, measles remains a leading vaccine-preventable cause of child mortality in China [6]. This vaccine schedule was replaced by a routine measles-rubella vaccine at 8 months of age, followed by a measles-mumps-rubella vaccine at 18–24 months of age according to the recommendation from China’s Advisory Committee on Immunization Programs in 2005 [7]. In 2010, China adopted the World Health Organization (WHO) Supplementary Immunization Activity (SIA) [8], which recommends a measles-mumps vaccine for children aged from 8 months to 4 years old regardless of their prior vaccination status.

Several studies [9,10] have examined the effect of two doses of measles vaccine in southern areas of China, leading to increased public health activity and reduced measles morbidity. In contrast, the northwestern regions of China have comparatively greater inequities in medical service [11]. In some areas, only 53.6% were found to have a first measles vaccine during the recommended time period [12]. Consequently, this area ranked second in China for measles morbidity with an infection reported in 3.47 per 10,000 people in 2014 [13]. Hence, a greater understanding of the factors associated with measles immunity in northwest China is needed.

Several studies in different countries have indicated that age [14], geographic region [15], history of measles vaccination [16], migration history [17], and history of measles infection [18] are associated with seropositivity of the measles antibody. However, few studies have examined the effectiveness of the measles vaccination and predictors for the measles antibody among children in northwest China. This study aimed to identify the social determinants of measles seropositivity and the effectiveness of SIA among children aged 2 to 4 years old in a large county of northwest China.

## 2. Materials and Methods

### 2.1. Study Design

A cross-sectional study was conducted among 755 healthy children aged 2 to 4 years in all 25 administrative townships in Qian County from February to September in 2014. Participants with acute febrile illness and infections, and individuals who were on immunosuppressive therapy were excluded. There were 247 children from rural areas, 384 from urban areas, and 124 from suburban areas. We planned to recruit ten children in every age group (2 years, 3 years, and 4 years) from each town. The starting point for participant contact was a randomly selected household in each town based on the house’s county register number. Selection then continued with next nearest household until ten eligible children were obtained. Using this process, we enrolled 755 children, slightly higher than our target of 750.

In each town, face-to-face interviews were conducted with parents or guardians who had children aged 2–4 years old. A questionnaire was administered by the staff to collect socio-demographic information, including the children’s gender, age, district type (rural, suburban, or urban), the mother’s educational level, and history of measles infection. Data on vaccination was taken directly from the town’s medical records.

Informed written consent was obtained from a parent or guardian of the participating children. Confidentiality was ensured by assigning anonymous study numbers to the questionnaires and samples before data entry and analysis. The study protocol was approved by the Biomedical Ethics Committee of Xi’an Jiaotong University Health Science Center (Code: 2013-279).

### 2.2. Laboratory Testing

Samples of 20 uL peripheral blood were collected via the ring finger from all subjects, centrifuged to isolate the sera, and frozen at −20 °C. Qualitative determination of immunoglobulin G (IgG) to measles in serum specimens was detected by using enzyme-linked immunosorbent IgG assays (ELISA) at the Center for Disease Control and Prevention in Qian County (kits were provided by the National Institute for Viral Disease Control and Prevention of Chinese CDC, Beijing, China). A determination of seropositivity was based on an IgG antibody titer of ≥1:200; a titer of <1:200 was considered seronegative according to the Standard GB15983-1995 guidelines of China [19].

### 2.3. Statistical Analysis

Data management and statistical analysis were performed using SPSS (Statistical Product and Service Solutions) for Windows (version 22.0) (IBM, Armonk, NY, USA). The Pearson’s Chi-square test was used for univariate analysis including gender, age, district type, maternal education level, maternal history of measles infection, and vaccine dose. Crude and adjusted odds ratios (OR) were obtained from logistic regression models adjusting for measles vaccine dose, district type, mother’s education, and maternal history of measles infection. In the logistic regression, the collinerarity between factors was solved by deleting the subordinate variables [20]. Statistical significance was set at *p* < 0.05; all tests were two-sided.

## 3. Results

### 3.1. Seroprevalence of Measles Antibody in Qian County

Overall, the measles antibody seroprevalence was 91.13% (95% CI: 89.52–92.83). The highest seroprevalence was observed in the towns of Guantou and Chengguan; Dayang had the lowest (Table 1).

### 3.2. Demographic Characteristics of the Children

The highest seroprevalence was observed in the 4 years age group (Table 2). Age (χ^2^ = 48.245, *p* < 0.001), maternal education level (χ^2^ = 8.738, *p* = 0.033), and vaccine dose (χ^2^ = 251.756, *p* < 0.001) were significantly correlated with seroprevalence (Table 2). The seroprevalence of children 2 and 3 years of age was significantly lower than that observed in the 4 years age group. No significant differences were found in seroprevalence with respect to gender, district type, or maternal history of measles infection.

### 3.3. Predictors for Seroprevalence among Children

We detected collinearity between age and measles vaccine dose (the condition index value was 11.37 and 16.12), so we removed the subordinate factor of age while analyzing by logistic regression to solve for it. Children vaccinated with two doses and three doses had 11.3 (95% CI: 4.0–32.2) and 685.5 (95% CI: 152.1–3089.1) times the odds of seropositivity of the measles antibody, respectively, compared with those receiving only one dose. Compared with children whose mother’s highest education was primary school, seroprevalence was higher in children whose maternal education was high school (adjusted OR: 2.2, 95% CI: 1.4–6.9) or college/university (adjusted OR: 2.6, 95% CI: 1.1–8.6). Neither district type nor maternal history of measles were significantly associated with seropositivity of the measles IgG antibody (Table 3).

## 4. Discussion

The results of our study showed 91.13% measles seropositivity among healthy children, which is below the 95% level recommended by the WHO [21]. Measles seropositive prevalence varies by country, with observed IgG levels of 89.5%, 98.2%, and 87.7% in Germany [22], United Arab Emirates [23], and Italy [24], respectively. In addition, the seroprevalence of the measles antibody in northwest China was lower than in other Chinese regions, such as Jiangsu (97.6%) [9], Tianjin (97.5%) [25], and Zhejiang (96.5%) [10] in southern China. The regional variation in measles seropositivity, both within China and across nations, might be due to differences in the design of early childhood immunization programs in each region [26] and inherited differences among various populations [27].

The higher seroprevalence of the measles antibody in children who received multiple doses of measles vaccine in our study is consistent with the results of previous studies [8]. As expected, multiple vaccine doses counter the natural decrease in immunity over time [21,28]. The seroprevalence in children that were administered one dose was far lower than in those receiving two doses. In some cases this may reflect a failure of the single dose because of vaccine degradation during storage or transport, rendering it less effective [29]. The primary vaccine failure rate was estimated to be 2–10% [30,31,32], but the secondary vaccination failure in those who developed seroconversion was shown to be considerably lower (0.2%) [33]. Furthermore, the first dose of the measles vaccine was administered at 8 months in China; vaccines given at that age may be nullified by maternal antibodies [34]. The seronegative rate was 24.5% in children administered two doses, which may be due to the delayed vaccine. Lower socioeconomic status, challenges in providing medical care, and poor dissemination of medical information may contribute to lower vaccine coverage in China [15].

In our study, the prevalence of antibodies increased with age, which is consistent with previous findings [16,35,36]. This might be due to the vaccination schedule or changes in maturing immune systems [26]. A study in Pakistan found that the levels of the measles-specific IgG antibody increased from 60.0% to 84.62% in children aged 9 months to 5 years [36]. Those IgG levels are lower than those in the present study, which may be related to the poor living conditions or nutritional status in the Pakistani sample. A study in Korea found that among children aged 1–3, 4–6, 7–9, and 10–12 years, the seropositive prevalence increased with age: 78.6%, 87.4%, 87.6%, and 92.7%, respectively, which was more in line with our results. The greater similarity of those results to ours may be related to the similar vaccination schedule or similar degrees of national development [37]. Nonetheless, children aged 4 years old was the only group that met the WHO target of >95% seropositivity in our study. This might be attributable to the implementation of SIA from 2010, which led to many children having three doses. Overall, routine vaccination was not satisfactory. Therefore, SIA appears to be an important way to eliminate measles.

The seroprevalence of measles antibodies was higher in children with highly educated mothers in the present study, which is consistent with previous studies [16,38,39]. A study in Pakistan showed an increasing trend of seropositivity with the mother’s education levels among children aged 12–59 months [16]. Another study in Bolivia found that seroprevalence of the measles antibody was higher in children with highly educated parents [38]. Consistent with those studies, a study in Istanbul showed that the vaccination rate was lower in children whose mother’s education level was primary school (89.8%) compared with those completing secondary school or higher (93.3%) [39]. Thus, efforts to decrease measles morbidity may need to be targeted to areas known to have lower levels of adult education.

There was no significant association between the district type and seropositivity of the measles antibody in our study. In China, the vaccination rate was lower in rural areas than urban areas according to the national Expanded Program on Immunization data [40]. A previous study conducted in 2008 in southern China found lower measles seropositivity in rural areas [15]. In our study, the seroprevalence was lower in rural areas than that in urban areas, but differences were not statistically significant. A weak association based on rural vs. urban areas in China may be related to the improvement of the rural primary health care service system and the implementation of SIAs from 2010. In addition, no significant difference was observed in the seropositivity of the measles antibody according to gender or maternal measles history, results that are consistent with those of previous studies [18,28,41].

Our study has some limitations. First, we relied on the mothers’ recall of measles history rather than medical records, which may have introduced differential or non-differential misclassification of exposure. Second, this study was conducted in a sample representing a northwestern district in China, so we cannot necessarily generalized our findings to the whole country. Moreover, our data are also 3 years old, and some changes in seroprevalence may have occurred since our data were collected.

## 5. Conclusions

The average seroprevalence of IgG to measles was 91.13% in the present study, which suggests an insufficient immunity among children aged 2–4 years old in northwest China. Our finding that seroprevalence was influenced by vaccine dose and the mothers’ education level suggests possible means to reduce measles morbidity.

## Figures and Tables

**Table 1:** Seroprevalence of the measles antibody in Qian County, China.

Region	Sample Size	Seroprevalence of Measles Antibody *n* (%)
Dayang	32	25 (78.13)
Tiefo	30	26 (86.67)
Xuelu	30	26 (86.67)
Jiangcun	31	27 (87.10)
Xinyang	31	27 (87.10)
Linping	31	27 (87.10)
Wudian	30	27 (90.00)
Daqiang	30	27 (90.00)
Zhugan	30	27 (90.00)
Lingyuan	31	28 (90.32)
Malian	29	27 (93.10)
Fengyang	30	28 (93.33)
Changliu	30	28 (93.33)
Liangcun	30	28 (93.33)
Moxi	30	28 (93.33)
Yanghan	30	28 (93.33)
Zhoucheng	30	28 (93.33)
Wangcun	30	28 (93.33)
Shiniu	30	28 (93.33)
Yangyu	30	28 (93.33)
Qianling	30	28 (93.33)
Liangshan	30	28 (93.33)
Yanghong	30	28 (93.33)
Guantou	30	29 (96.67)
Chengguan	30	29 (96.67)
Total	755	688 (91.13)

**Table 2:** Seroprevalence of the antibody to measles in association with demographic variables among 755 healthy children in Qian County, China.

Demographic Variables	Sample Size	Measles Antibody Seroprevalence *n* (%)	χ*^2^*	*p*
Gender				
Boy	425	384 (90.4)	0.718	0.397
Girl	330	304 (92.1)		
Age				
4 years	247	242 (98.0)	48.245	<0.001
3 years	251	237 (94.4)		
2 years	257	209 (81.3)		
District type				
Rural	247	223 (90.3)	1.942	0.379
Suburban	124	110 (88.7)		
Urban	384	355 (92.4)		
Mother’s education				
Primary school	190	165 (86.8)	8.738	0.033
Middle school	221	199 (90.0)		
High school	208	195 (93.8)		
Vocational College/University or higher	136	129 (94.9)		
Measles vaccine dose				
1	24	5 (20.8)	251.756	<0.001
2	188	142 (75.5)		
3	543	541 (99.6)		
Maternal history of measles infection				
Yes	34	31 (91.2)	0.648	0.723
No	683	621 (90.9)		
Unknown	38	36 (94.7)		

**Table 3:** Demographic and other predictors of measles antibody seropositivity among healthy children: a multivariable logistic regression model.

Variables		Crude OR	95% CI	*p*	Adjusted OR	95% CI	*p*
Measles vaccine dose	1 (reference)	-					
	2	11.7	4.1–33.2	<0.001	11.3	4.0–32.2	<0.001
	3	1027.9	187.3–5640.4	<0.001	685.5	152.1–3089.1	<0.001
District type	Rural (ref)	-					
	Suburban	0.8	0.4–1.7	0.637	0.9	0.7–1.3	0.713
	Urban	1.3	0.7–2.3	0.339	1.1	0.8–1.3	0.341
Mother’s education	Primary school (ref)	-					
	Middle school	1.4	0.7–2.5	0.309	1.1	0.8–1.4	0.802
	High school	2.3	1.1–4.6	0.019	2.2	1.4–6.9	0.006
	Vocational College/University or higher	2.8	1.2–6.7	0.017	2.6	1.1–8.6	0.037
Maternal history of measles disease	Yes (reference)	-					
	No	1.0	0.3–3.3	0.960	0.8	0.3–2.7	0.923
	Unknown	1.7	0.3–11.1	0.553	2.4	0.8–16.2	0.477

## Abbreviations

The following abbreviations are used in this manuscript:

IgG	Immunoglobulin G
WHO	World Health Organization
MMR	Measles, Mumps, and Rubella
SIA	Supplementary Immunization Activity
OR	Odds Ratio
CI	Confidence Interval
